# Single and dual task gait training in people with Parkinson's Disease: A protocol for a randomised controlled trial

**DOI:** 10.1186/1471-2377-11-90

**Published:** 2011-07-27

**Authors:** Sandra G Brauer, Marjorie H Woollacott, Robyn Lamont, Sandy Clewett, John O'Sullivan, Peter Silburn, George D Mellick, Meg E Morris

**Affiliations:** 1 School of Health and Rehabilitation Sciences, The University of Queensland, Brisbane, QLD 4072 Australia; 2 Department of Human Physiology, The University of Oregon, Eugene, OR 97401 USA; 3 Royal Brisbane Clinical School, School of Medicine, The University of Queensland, Brisbane 4072 Australia; 4 UQ Centre for Clinical Research, The University of Queensland, Brisbane, QLD 4072 Australia; 5 Eskitis Institute for Cell and Molecular Therapies, Griffith University, Nathan, QLD 4111 Australia; 6 Melbourne School of Health, The University of Melbourne, Melbourne, VIC 3010 Australia

## Abstract

**Background:**

Difficulty performing more than one task at a time (dual tasking) is a common and disabling problem experienced by people with Parkinson disease (PD). If asked to perform another task when walking, people with PD often take shorter steps or walk more slowly. Currently there is uncertainty about whether clinicians should teach people with PD to avoid dual tasking or whether they should encourage them to practice dual tasking with the hope that practice will lead to enhanced performance. This study will address this issue by comparing single to dual task gait training.

**Methods and design:**

A prospective randomised clinical trial is being conducted. Sixty participants with idiopathic PD will be recruited, provided they score I-IV on the modified Hoehn and Yahr (1967) scale, and fulfil other inclusion criteria. Participants will be randomly allocated to either a single or dual task gait training group. Both groups will receive 12 hours of walking training over 4 weeks. The single task group will undertake gait training with cueing strategies to increase step length. The dual task group will train to improve step length when walking and performing a variety of added tasks. Both groups will receive a tailored home program for 6 months. Blinded assessors will conduct four assessments: two baseline assessments, one post intervention and one at 6 months follow-up. The primary outcome measure will be step length when dual tasking over 8 m. Secondary outcome measures include: spatiotemporal gait parameters when walking under single and dual task conditions, measures of executive function, the timed up and go test, measures of community mobility, and quality of life. All analyses will be based on intention to treat principle.

**Discussion:**

This trial will examine the immediate and longer term effect of dual task walking training as compared to single task training in people with idiopathic PD, at the impairment, activity, and participation levels. It has the potential to identify a new intervention that may improve and maintain walking beyond the laboratory. The results of this trial will provide guidance for clinicians in the development of walking training programs for people with PD.

**Trial Registration:**

ACTRN12609000791235

## Background

Over 4 million people worldwide are estimated to be diagnosed with Parkinson disease (PD), with this projected to double within the next 20 years [1]. More than half of community dwelling adults living with PD experience gait disturbances that are associated with increased disease severity, disability, poor quality of life [2] and caregiver strain[3,4]. Falls are a common complication of PD, with between 50 and 68% of people with PD falling each year [5-8], with most falls reported to occur when walking [9]. Medications such as levodopa, provide the cornerstone of treatment of gait disturbances in PD, however these become less effective over time for some symptoms of PD including gait disturbance. There is growing evidence that strategy training, whereby internal or external cues are used to direct a person's attention to normalise their gait deficits, is effective in augmenting drug therapy to improve gait in the short term [10]. Internal cues involve the person directing their attention to consciously think of improving the deficit (e.g. thinking 'big steps'). External cues aim to normalise the gait deficit and often include verbal cues (e.g. saying 'big steps'), visual cues (e.g. lines on the floor at the desired step length) [11], or auditory cues (e.g. metronome to normalise cadence) [12]. The majority of this evidence is based on laboratory-based training [10] with low sample sizes, and measures of immediate effects of therapy. The longer term effect of strategy training is not yet clear.

For gait to be functional in daily life both within the community and home, people need to be able to dual task. This could involve thinking when walking or maintaining balance when holding an object. It is well known that performing an added task interferes with postural stability in older adults (termed dual task interference) [13], particularly in those adults with impaired balance [14-16].

It is well established that when asked to perform a concurrent task when walking, people with PD demonstrate reduced gait velocity, step length [11,17-20], increased stride to stride variability [21] and more freezing episodes [22,23] than when walking alone. These gait disturbances are also known falls risk factors [6]. An underlying rationale proposed for dual task interference in PD is that when required to perform two tasks at the same time, one runs through the frontal cortical regions and is under conscious control while the other is controlled by the defective basal ganglia. The task controlled by the frontal lobes is typically performed with normal speed and amplitude whereas the task controlled by the basal ganglia may show errors and be under-scaled in speed, amplitude and force [11].

Despite laboratory studies showing the serious effects of dual task interference on the spatiotemporal gait variables in PD, to date there have been few investigations of the effects of training on dual task interference during locomotion in PD. A pilot study of 90 minutes of multiple task training in 5 people with mild to moderate Parkinson Disease reported that participants reported low levels of fatigue, difficulty and anxiety and high levels of confidence [24]. A recent study has also demonstrated that people with PD can improve their ability to dual task when walking with training [25], however it only investigated the immediate effects of a 20 minute gait training session.

The primary aim of this randomised controlled trial (RCT) is to determine the short and longer-term effects of dual-task training compared to single task training during gait on walking performance when dual tasking in people with PD. In addition, we will determine the impact of the training programs on gait impairment, executive function, community mobility and quality of life.

## Methods

### Design

**Figure 1:**
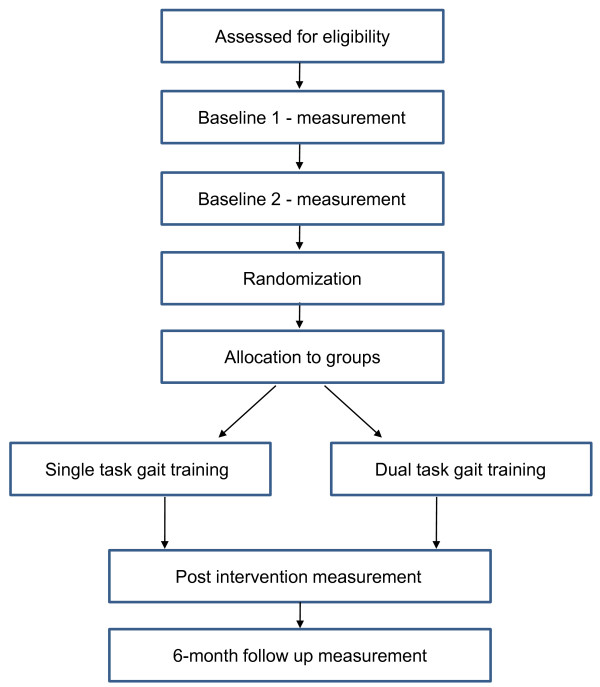
**Trial design**.

A prospective, single blinded two group randomised clinical trial with 6 month follow up of 60 people with PD living in the community setting will be conducted (Figure 1).

### Location and setting

We plan to recruit 60 people with PD, through Parkinson's Queensland Inc., a support organisation for people with PD, neurologists who specialise in Movement Disorders, and from the Queensland Parkinson's Project - a research study register of over 3000 community dwelling Queenslanders, recruited since 2005, who have agreed to participate in research into Parkinson's Disease and related disorders. Assessment will be undertaken at the Princess Alexandra Hospital, Brisbane, and training undertaken at St Andrew's War Memorial Private Hospital, Brisbane.

### Population

To be included in the study participants must meet the following criteria: (i) aged 18 years or older; (ii) a diagnosis of idiopathic PD using the UK Brain Bank criteria; (iii) able to walk 100 m independently with or without gait aids; (iv) rated stage I-IV on Hoehn and Yahr disability scale [26]; and (v) report reduced step length or slowed gait speed, confirmed by clinical examination. Participants will be excluded if they have neurological conditions other than PD, musculoskeletal or cardiopulmonary conditions that affect the ability to safely walk, had surgery for PD such as deep brain stimulation, score < 24 on the Mini-Mental Status Examination (MMSE) [27]; or sensory system pathology affecting walking or communication (e.g. blindness, deafness). All volunteers will be screened by a physiotherapist.

### Randomisation and blinding

Concealed randomisation will be prepared by an offsite investigator who is not involved in recruitment, intervention or data collection using a computer generated random number sequence. Consecutively numbered, randomly ordered opaque envelopes containing group allocation in 1:1 ratio will be opened consecutively after baseline assessment by the physiotherapists implementing the intervention.

Research assistants who enrol participants, and conduct pre, post and follow-up assessments will be blinded to group allocation throughout the study. Participants will not be informed of group allocation, however they may become aware of group allocation due to the nature of the intervention. Participant coding will not refer to group and participants will be instructed not to divulge information regarding their intervention to the research assistants undertaking the assessments. Treating physiotherapists will not be blind to group. To control expectancy effects for treating physiotherapists and patients it will be explained that it is not yet determined which therapy is more effective.

### Intervention

#### Single task training

Participants in the single task control group will participate in a 12-session program administered for 40-60 minutes each session, typically undertaken 3 times per week for 4 weeks. The intervention will be performed by a trained physiotherapist on a one-on-one basis, with training commencing at the patient's self-reported optimal 'ON' period, often 1 hour post medication. The single task group will receive an individually-progressed program of gait training aimed at improving step length via repeated practice of straight line walking, turning, obstacle negotiation and challenging gait tasks such as increasing speed and altering surface challenges. The classes will be performed in a hospital environment. A weekly guide for progression of task type and difficulty will be used. External cues to increase step length when walking will be used when needed and may include verbal, visual or auditory approaches. Participants will be instructed to concentrate on their walking performance during training. When training, care will be taken to ensure instructions are not performed when walking to avoid dual tasking during this intervention. A home program will be progressively integrated from week 2 with participants asked to continue with this program for 6 months. This will include a walking program, and a range of balance, strengthening and postural exercises particular to the patient, but chosen from a set of potential exercises. On completion of the intervention, participants will have a daily calendar in which to record their home program, and will receive monthly reminder follow-up telephone calls.

#### Dual task training

Participants in the dual task training group will also participate in a 12-session program administered for 40-60 minutes each session, typically undertaken 3 times per week for 4 weeks. As per the single task training group, the intervention will be undertaken by a trained physiotherapist on a one-on-one basis, with training commencing at the patient's self-reported optimal 'ON' period, often 1 hour post medication. The dual task gait training program will aim to improve step length under dual task conditions, that is, when concurrently performing added cognitive or motor tasks. Participants will undertake repeated practice of walking aiming to improve step length using external cueing techniques as outlined above, progressing to internal concurrent cueing of appropriate step length. The gait tasks undertaken will be progressed from simple to more complex tasks as outlined for the single task group.

In addition, a variety of added tasks will be progressively integrated into the training program. These include tasks such as listening, speaking, conversing, generation of simple and complex lists, language, calculation and motor tasks increasing in complexity. Tasks will include those designed to reflect functional everyday activities, such as carrying bags, getting keys out of a pocket, counting money, recalling directions or making a shopping list. Complexity will be progressively integrated as more complex tasks result in greater dual task interference with gait in people with PD [17,23,28,29]. Tasks involving maths and calculation will also be included as added language and calculation tasks can differentially impact gait performance in people with PD [20]. If able, participants will be progressed to performing increasingly complex cognitive tasks while concurrently walking. These may include visual spatial planning tasks (e.g. tell me how to get from here to reception), response inhibition tasks (e.g. complete the phrase without saying ...), or tasks integrating both language and calculation components (e.g. if Saturday is the 8^th^, what is the date the following Thursday?). Motor tasks such as carrying and manipulation will also be included as added tasks, as some studies have reported that added cognitive and motor tasks can both impact gait performance in people with PD [23]. These may be combined with cognitive tasks (e.g. getting a certain amount of money out of a wallet when walking). A weekly guide for progression of task type and difficulty will be used and programs will be individually-progressed.

Instructional set will commence with fixed priority training, whereby attention is equally shared between the walking and added task, but will aim to progress to variable priority training, whereby the proportion of attention directed to the gait and added task is varied from repetition to repetition [30]. For example, on one walking pass, a participant will be asked to concentrate mainly on taking big steps when also performing a counting task. In the next pass, they will be asked to concentrate mainly on the added task, trying to count as quickly and accurately as possible. Variable priority training has been shown to result in better balance during dual task gait conditions in older adults with balance impairment than fixed priority training [30], so this approach will be gradually introduced.

### Outcome measures

The primary outcome measure will be step length when dual tasking over 8 metres. Reduced step length has been shown to be one of the primary gait deficits in people with PD [31]. Participants will walk an unobstructed 10 metre path over an 8 metre GAITRite^® ^electronic walkway at a comfortable pace. At the same time, they will be asked to perform an added spoken task, including saying as many words as possible beginning with certain letters, termed the controlled oral word association test (words), or counting backwards by 3s beginning with a number between 50 and 100 (count). Both these tasks have been used to demonstrate altered gait in people with PD when dual tasking [20,25]. Step length is calculated by the GAITRite system, with the mean value used in analysis. The GAITRite^® ^system has demonstrated sensitivity to change in gait parameters in people with PD [32] and has been used as a gold standard comparator when investigating gait under dual task conditions in people with PD [33]. Assessment will be performed during the patient's self-reported optimal 'ON' period, often 1 hour post medication.

Secondary outcome measures include: spatiotemporal gait parameters when walking under single and dual task conditions, cognitive function, functional gait performance, community mobility, and quality of life. Spatiotemporal gait parameters other than step length will be recorded when walking at a comfortable pace over 10 metres under both gait only (single task) and the two dual task conditions detailed previously. These will include gait speed (m/s), cadence (steps/min), stride length (m), step length coefficient of variation (CV) and double support time (s).

Cognitive domains to be tested by a neuropsychologist include: executive function, attention, visual perception and processing speed. Tests have been selected based on their ability to assess the required domains, and their ability to detect deficits in the Parkinson Disease population [34]. Tests include the Trail making A & B Tests [35]; the Stroop colour-word interference test [36], and a Digit span test [37].

Functional gait performance will be measured using the Timed Up and Go (TUG) test with added motor and cognitive tasks [38]. Participants will be asked to walk as quickly as they safely can under all conditions and will be permitted to use their usual gait aid. A six minute walk test will be completed to quantify walking capacity. This test has demonstrated high reliability in people with PD (ICC = 0.95) [39].

Community mobility will be quantified using a questionnaire based on a self-report tool designed for community-dwelling older adults [40]. Activity will be measured over 3 days using an ActivPAL[41]. Health-related quality of life will be measured using a generic health-related quality of life measure, the EuroQuol (EQ-5D) [42] and the PDQ-39, which is specific to the Parkinson Disease population [43].

Several measures will be taken to characterise participants at each assessment session. Severity of PD will be categorised using the Hoehn and Yahr scale [26] and the Unified Parkinson's Disease Rating Scale (UPDRS) motor subsection III [44]. Freezing will be characterised using the Freezing of Gait questionnaire [45] and confidence will be measured using the ambulatory self confidence questionnaire [46]. General demographic information to be recorded will include: age, disease duration, medical history, number of falls in previous year, mobility aid use, medication type and dosage. Medication will be documented in detail at every assessment timepoint.

### Data analysis

Data analysis will be performed on an intention-to-treat basis using an alpha level of 0.05. Descriptive statistics will be used to ensure comparability of scores between groups at baseline, to describe performance at each phase and to test whether the assumptions for use of parametric statistics have been met. If the assumptions for F or t-tests are violated, equivalent non-parametric statistics will be utilized. The main hypothesis will be tested using General Linear Models (repeated measures analysis of variance, ANCOVA), in a 2 group (single task, dual-task) × 4 phase (baseline 1, baseline 2, post intervention, follow up) model. This will be followed by between-groups planned comparisons using the t-statistic. All secondary outcomes will be analysed in a similar manner. Disease duration and age will be used as co-variates in all analyses. Data will be analysed using SPSS.

### Sample size

Large standardized effect sizes (e.g. 0.1-0.5 m stride length; 10-25 m/min gait speed) shown in previous experiments where there were 2 active interventions to improve gait in people with PD using strategy training [11] (Cohen's d >5) led to the prediction that a sample size of 30 in each group will yield power in excess of 99% with α = 0.05 for step length and speed. Previous studies with similar populations of people with PD (Morris [11,28,29,31] et al., indicate that attrition is typically in the range of 5-8%. Even if the sample diminished by 2 in each group, the power would still exceed 95%.

### Ethics approval

The study protocol has been approved by the University of Queensland Medical Research Ethics Committee (MREC ID: 2007001631), the Princess Alexandra Hospital Ethics committee (ID: 2009013), the Uniting Care Health Human Research Ethics Committee (ID: 0911).

## Discussion

This trial will be the first to compare the effect of dual task training when walking with single task (usual strategy gait training) in people with PD. Currently it is not clear whether training dual tasking when walking should be advocated in people with PD. It will measure the effect on not only gait performance, but also on other indicators of impairment, activity and participation both immediately following training, and for a follow up period of 6 months. This data will assist in determining the impact of physiotherapy gait training for people with Parkinson Disease who are still mobile and living in their own homes. It will be one of the first studies to monitor the effect of this type of intervention on community mobility disability in the PD population, and to determine transference to activities in daily life.

Findings from this study will provide insights into the effects of practice on dual-task performance in people with PD. It will generate new knowledge regarding optimal principles of training to enable people with PD to overcome debilitating dual task interference during rehabilitation. The information gained from this project has the potential to change the way gait training is structured, and will provide clear evidence as to whether dual task training should be advocated in people with PD. Our results are likely to have wider implications for training other populations who commonly demonstrate dual task interference with postural tasks such as elders [14,15] and people with brain injuries [16] as it is expected that the principles underpinning the design of the training program will be transferable to these populations.

## List of abbreviations

PD: Parkinson's Disease; RCT: Randomised Controlled Trial; MMSE: Mini Mental Status Examination; CV: Co-efficient of Variation; TUG: Timed Up and Go; ICC: Intraclass Correlation Coefficient; EQ-5D: EuroQuol; PDQ-39: Parkinson's Disease Questionnaire-39; UPDRS: Unified Parkinson's Disease Rating Scale; ANCOVA: Analysis of Covariance; SPSS: Statistical Processes for the Social Sciences.

## Competing interests

The authors declare that they have no competing interests.

## Authors' contributions

SB conceived the idea for the study. SB, MM and MW all contributed to the research design and obtained funding for the study. SB, MM, MW, SC and RL contributed to the design of the study, intervention and outcome measures. JO, PS and GM were involved in patient recruitment. SB was principally responsible for the drafting of the manuscript. All authors assisted in editing the final submitted manuscript. All authors have read and approved the manuscript.

## Pre-publication history

The pre-publication history for this paper can be accessed here:

http://www.biomedcentral.com/1471-2377/11/90/prepub
